# Feasibility of a randomized controlled trial to evaluate the impact of decision boxes on shared decision-making processes

**DOI:** 10.1186/s12911-015-0134-x

**Published:** 2015-02-25

**Authors:** Anik MC Giguere, Michel Labrecque, France Légaré, Roland Grad, Michel Cauchon, Matthew Greenway, R Brian Haynes, Pierre Pluye, Iqra Syed, Debi Banerjee, Pierre-Hugues Carmichael, Mélanie Martin

**Affiliations:** Department of Family and Emergency Medicine, Research Center of the CHU de Quebec, Saint-Francois d’Assise Hospital, Laval University, 10 rue de l’Espinay, D6-730, Quebec City, QC G1L 3L5 Canada; Department of Family Medicine, McGill University, Herzl Family Practice Centre, 3755 Cote Sainte Catherine, Montreal, QC H3T 1E2 Canada; Department of Family Medicine, McMaster University, 118 Lake Street, St. Catharines, ON Canada; Department of Clinical Epidemiology and Biostatistics, McMaster University, 1280 Main Street West, CRL-125, Hamilton, ON L8S 4K1 Canada; Department of Medicine, DeGroote School of Medicine, McMaster University, 1280 Main Street West, CRL-125, Hamilton, ON L8S 4K1 Canada; Department of Family Medicine, McGill University, 5858 Côte-des-neiges, 3rd Floor, Suite 300, Montreal, QC H3S 1Z1 Canada; The University of Toronto, Faculty of Medicine, 1 King’s College Circle, Medical Sciences Building (Rm. 2109), Toronto, ON M5S-1A8 Canada; Research Centre for Excellence in Aging, CHU de Quebec, Saint-Sacrement Hospital, 1050 chemin Ste-Foy, Québec, Québec G1S 4L8 Canada; Department of Family and Emergency Medicine, Laval University, Pavillon Ferdinand-Vandry, 1050 avenue de la Medecine, Quebec City, Quebec G1V 0A6 Canada

**Keywords:** Evidence-based practice, Continuing professional education, Shared decision making, Risk communication, Patient-centered care, Counseling, Clinical topic summary, Decision support, Knowledge translation

## Abstract

**Background:**

Decision boxes (DBoxes) are two-page evidence summaries to prepare clinicians for shared decision making (SDM). We sought to assess the feasibility of a clustered Randomized Controlled Trial (RCT) to evaluate their impact.

**Methods:**

A convenience sample of clinicians (nurses, physicians and residents) from six primary healthcare clinics who received eight DBoxes and rated their interest in the topic and satisfaction. After consultations, their patients rated their involvement in decision-making processes (SDM-Q-9 instrument). We measured clinic and clinician recruitment rates, questionnaire completion rates, patient eligibility rates, and estimated the RCT needed sample size.

**Results:**

Among the 20 family medicine clinics invited to participate in this study, four agreed to participate, giving an overall recruitment rate of 20%. Of 148 clinicians invited to the study, 93 participated (63%). Clinicians rated an interest in the topics ranging 6.4-8.2 out of 10 (with 10 highest) and a satisfaction with DBoxes of 4 or 5 out of 5 (with 5 highest) for 81% DBoxes. For the future RCT, we estimated that a sample size of 320 patients would allow detecting a 9% mean difference in the SDM-Q-9 ratings between our two arms (0.02 ICC; 0.05 significance level; 80% power).

**Conclusions:**

Clinicians’ recruitment and questionnaire completion rates support the feasibility of the planned RCT. The level of interest of participants for the DBox topics, and their level of satisfaction with the Dboxes demonstrate the acceptability of the intervention. Processes to recruit clinics and patients should be optimized.

**Electronic supplementary material:**

The online version of this article (doi:10.1186/s12911-015-0134-x) contains supplementary material, which is available to authorized users.

## Background

Shared Decision Making (SDM) is a process that involves clinicians and patients making joint decisions based on the best available evidence on the benefits and harms of all available options, and on the patient’s values and preferences [1]. To date, researchers have mostly studied two strategies to implement SDM in clinical practice: patient-mediated interventions (mostly patient decision aids) [2] and educational meetings for clinicians [3].

As participation in educational meetings can sometimes be challenging for busy clinicians [4], we created the “decision box” (DBox), a two-page research-based evidence summary that clinicians can receive by email or access online, and use in printed or electronic format at their own pace. Dboxes are continuing professional development tools meant to prepare clinicians for shared decision making. They work by helping clinicians recognize equipoise and the need to share a decision with the patient, and by providing the information about the risks and benefits of all the options [5]. Following exposure of clinicians to a series of Dboxes, we expect that they will transmit the educational information from Dboxes to patients, which represents one level of outcomes of continuing education [6]. The DBoxes were not meant to be shown to patients, but our recent study suggests that the DBox could be distributed to clinicians and staff playing an important role in the delivery of simpler tools for patients, such as patient decision aids [7].

We engaged in a series of studies to develop preliminary DBox templates and prototypes [8], to adapt and standardise the content and format of the DBoxes to users’ needs and preferences [8,9], and to study the barriers to using the educational information from DBoxes in practice, and tailor them to the identified barriers [10,11]. The next step was thus to evaluate the feasibility of conducting a clustered randomized controlled trial (RCT).

### Objectives of the study

Feasibility studies are generally used to evaluate processes, resources, management and/or scientific issues [12]. In preparation for a RCT to evaluate the impact of DBoxes, our feasibility study objectives were to assess (i) the acceptability of the planned intervention to clinicians (level of interest of participants for the DBox topics, and their level of satisfaction with the DBoxes), and (ii) the feasibility of study processes, with respect to time, resources and management. We also aimed to assess the feasibility of the recruitment of clinicians and patients and the variability of the primary outcome to calculate the required sample size for the subsequent RCT.

### Design of a trial to evaluate the impact of the DBox on decision making processes

**Figure 1:**
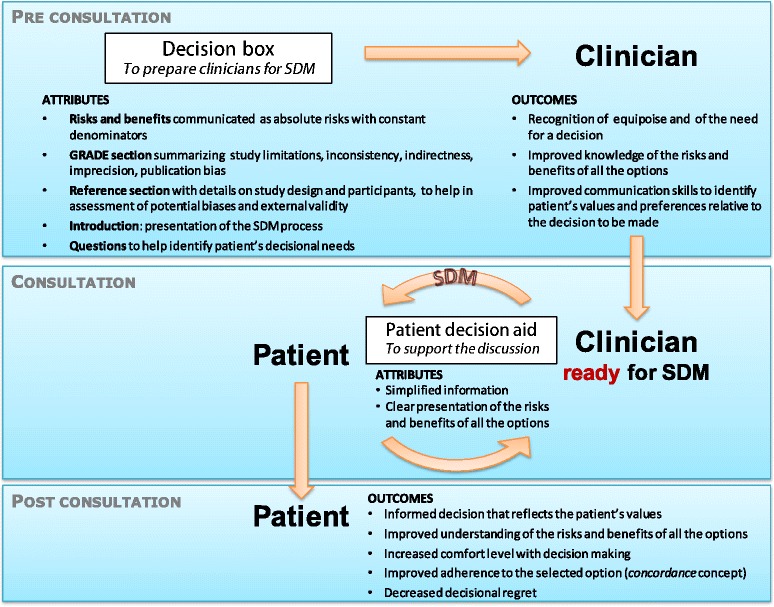
**Theoretical model of the decision box.** Theoretical model of the decision box and the mechanisms by which it supports shared decision making (SDM), including its specific aim, the attributes designed to reach this aim, and the outcomes to evaluate whether the aim was reached.

There is a lack of consensus on the best evaluation approach to test the effectiveness of SDM interventions, although it is generally agreed that their ultimate goal should be to facilitate informed, preference-sensitive decision making by the patient and clinicians [13]. Charles et al. emphasized how goal setting activities should drive measurement activities and not the other way around [14,15]. They proposed three concepts to judge the relevance of a SDM intervention: having a clear and explicit rationale, a clear definition of the construct, and a theoretical basis for making hypotheses on how the intervention might produce a particular outcome [14]. Hence, based on literature reviews [8,16] and on our previous empirical work on the Dbox [7,17], we designed a theoretical model that clarifies the DBox’s specific aims, its attributes to reach each aim, and the outcomes to evaluate whether the aim was reached (Figure 1). This model led to the selection of outcomes for assessing the impact of SDM interventions.

With regards to our theoretical model (Figure 1), the planned RCT will assess the impact of DBoxes delivered to clinicians, compared to usual care, on patients’ involvement in decision-making during clinical consultation. The primary outcome of the planned RCT, patient involvement in decision-making, will be measured using the 9-item Shared Decision Making Questionnaire (SDM-Q-9) [18].

## Methods

### Study design

This feasibility study was an add-on to a larger study on the barriers and facilitators to the implementation of the DBoxes in primary healthcare clinics in Canada, reported elsewhere [7,10]. It included a single experimental group of participants from several clinics who were exposed to the intervention. Measurements were made only after the implementation of the intervention.

### Intervention

The intervention consisted of eight evidence-based DBoxes on common primary care diagnostic, therapeutic and preventive interventions (Table 1) written in both French and English, and a website supporting the DBoxes (http://www.decisionbox.ulaval.ca) that comprised a brief web-based tutorial to introduce the DBox, and educational material on patient counseling and on the GRADE ratings of the quality of evidence integrated in the DBoxes. Each week, the participating clinicians received one DBox by email for a total of eight weeks. We asked clinicians to read the DBoxes and then complete a web-questionnaire for each. The Dboxes all offered a main message to share the information provided in the DBoxes with their patients.

**Table 1:** **Clinical topics covered by each decision box and their order of delivery**

**Clinical topics covered by decision boxes** ***(abbreviation, used throughout the text to refer to each clinical topic)***	**Order of delivery**
Cholinesterase inhibitors to reduce the symptoms of Alzheimer’s disease *(ChEIs)*	1
Acetylsalicylic acid for primary prevention of cardiovascular disease *(ASA)*	2
The fecal occult blood test to screen for colorectal cancer *(FOBT)*	3
The serum integrated test to screen women for fetal trisomy 21 *(Prenatal)*	4
Statins for primary prevention of cardiovascular disease *(Statins)*	5
The BRCA1/2 gene mutation test to evaluate the risks of breast and ovarian cancer *(BRCA)*	6
Bisphosphonates to prevent osteoporotic fractures in postmenopausal women *(OSTEO)*	7
The prostate-specific antigen test to screen men for prostate cancer *(PSA)*	8

### Primary outcomes

Acceptability of the DBox intervention and feasibility of the planned RCT were the primary outcomes of the present study. In order for the intervention to be deemed acceptable, we determined *a priori* and arbitrarily that at least 80% of clinicians’ ratings should demonstrate a level of satisfaction towards the intervention of 4 or 5 out of 5. For feasibility, we set as criteria that: (i) we should recruit at least five primary healthcare clinics [10]; (ii) clinicians’ recruitment rate should reach at least 50%; (iii) at least 70% of the participating clinicians should complete half of the eight web-questionnaires, or more [10]; and (iv) four patients should be recruited per clinic per day – a recruitment rate observed in a previous study conducted in similar settings [19] – during one week, in order to reach an overall sample size of 100 patients.

### Healthcare clinic and clinician recruitment processes

Twenty family medicine clinics were invited to participate in this study. We initially contacted six primary healthcare clinics where the study investigators held professional contacts. We simultaneously invited 14 clinics where none of the investigators had any professional contact, in a radius of 40 km around the city of Hamilton (Ontario, Canada).

### Patient eligibility criteria and recruitment processes

Following an 8-week intervention period, the clinic’s staff distributed advertisements about the study to all patients attending the clinic, and informed them verbally that they could meet a research assistant at a predefined location within the clinic if they wanted more information. The research assistant invited the interested patients to the study and screened them as they left participating clinicians’ office. The patient inclusion criteria for the feasibility study are the same as those for the RCT, and comprise: (i) being 18+ years of age, (ii) consulting participating clinicians during the recruitment period (i.e. convenience sample), (iii) reporting having discussed one of the eight clinical topics covered in the decision support tools with their clinicians on the day of recruitment, and (iv) agreeing to participate in the study. Because we expected that clinicians communicate the DBox educational information to their patients without showing them the actual DBox, then we did not exclude patients who had not seen a Dbox. We offered monetary compensation (CAN $20) to patients who participated in the study interviews.

### Data collection and procedures

At study entry, participating clinicians signed an informed consent form and completed a questionnaire assessing their demographic and professional characteristics (age, gender, number of years of clinical practice). They also rated their interest in each of the eight DBox topics using a visual analogue scale ranging from 1 ("no interest") to 10 ("high interest"). The order of delivery of the eight DBoxes was pre-determined randomly by the principal investigator (AMCG), using opaque and sealed envelopes, and the same order of delivery was used in all the study sites (Table 1). For logistical reasons, the sites did not receive the intervention simultaneously, but instead started receiving the DBoxes, at one-week intervals. For each DBox received, clinicians were asked to complete a questionnaire containing a 5-point smiley-faces rating scale to assess global satisfaction with the DBox, and other measures described and reported elsewhere [7,10]. Questionnaire completion allowed assessing if participants actually read the DBoxes.

Participating patients completed the informed consent forms, and a self-report questionnaire to identify the type of health problem (s) discussed, who it was discussed with, which decision was made regarding this problem, and whether (or not) they saw a DBox during their clinical consultation. Among the eight topics covered by the DBoxes, if more than one was discussed, we asked the patient to identify the one that was discussed the most. The patient also answered questions to assess the decision making process during the consultation using the SDM-Q-9 instrument,[18] and their decisional conflict relative to this health problem with the DCS [20] (Additional file 1).

### Analysis

We calculated the proportion of recruited clinics among those invited. Clinician recruitment rate was calculated as the proportion of clinicians (i.e., physicians, residents and nurses) that participated in the study, out of all those that were invited. Patients’ recruitment rates were calculated in terms of the number of patients recruited per clinic per day.

We performed descriptive statistical analyses of all the answers to the questionnaires. We used a one-way ANOVA to test for significant difference in baseline interest for clinical topics, and a two-way ANOVA to test if the type of clinicians (nurse, resident, and physician) modulated the interest in clinical topic (factors: topic and types of clinicians).

For the answers to the web-questionnaires, we initially visually inspected a graph of the frequency of clinicians who completed the questionnaire. We also evaluated the proportion of questionnaires completed for each topic and tested if the completion rate was influenced by the order of delivery of the DBoxes.

We used patients’ ratings of SDM processes (SDM-Q-9) to calculate the power and sample size needed for the subsequent RCT planned to evaluate the effectiveness of the DBox to improve these outcomes. Sample size calculations were based on an unadjusted *T* test of standardized mean differences for the post-intervention comparison, assuming homogeneity of variances across experimental groups.

Approvals of ethics for this project were given by the research ethics committees of the Centre de Recherche du Centre Hospitalier Universitaire de Quebec (reference number #S11-12-143), Jewish General Hospital in Montreal (reference number #12-014), and McMaster University (reference number #11-550).

## Results

### Healthcare clinics’ recruitment

Among the 20 family medicine clinics invited to participate in this study, four agreed to participate in both phases of the study, giving an overall recruitment rate of 20%. Two additional clinics accepted to participate only in the first part of the study that consisted in receiving DBoxes and completing the web-questionnaires, as they were concerned that patient recruitment would lead to work overload. We recruited no patients in those two clinics.

The six recruited clinics were among those where the study investigators had previous professional contacts. Among the 14 family medicine clinics where none of the investigators had any professional relationship, eight declined participating to this study and six did not get back to the research coordinator despite a proper follow-up of all communications (whenever clinic staff mentioned that we should call another person, or at another time).

### Clinician recruitment and eligibility rates

**Figure 2:**
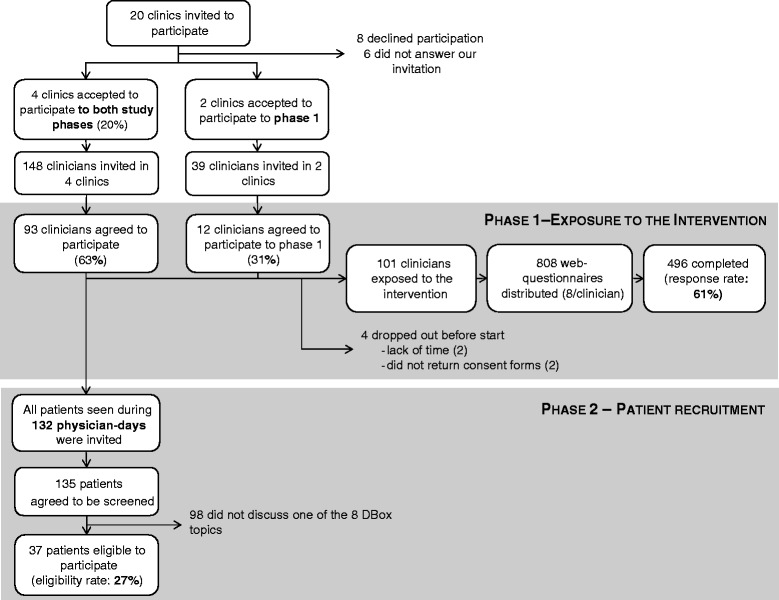
**Clinician recruitment, questionnaire completion rates, patient recruitment and eligibility rates.**

Overall, 93 clinicians from four clinics located in four cities accepted to participate in the two phases of the study, out of 148 that were invited, giving an overall recruitment rate of 63% (Figure 2). Of the 105 clinicians who accepted to participate to phase one, two dropped out (1.9%), as they realized during the course of the study that they would not have enough time to complete the web-questionnaires whereas two returned the consent forms after the start of the study and were thus not included, leaving 101 clinicians exposed to the intervention

Of those who completed the questionnaires, most were physicians (n = 94; 90%), among whom a third were residents (n = 30), and 11 nurses also participated (11%).

### Acceptability: interest for clinical topics and satisfaction towards the DBoxes

**Figure 3:**
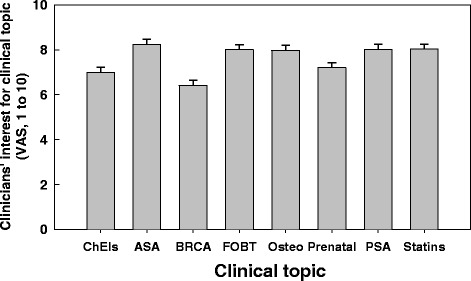
**Clinicians’ interest for the decision box clinical topics.** Clinicians’ interest for the decision box clinical topics measured using a visual analog scale at baseline (see Table 1 for the abbreviation legend).

At study entry, clinicians rated an interest for the DBox topics ranging from an average of 6.4/10 (standard error of mean = 0.2) for ‘BRCA’ (see Table 1 for the abbreviations) to 8.2/10 (standard error of mean = 0.2) for ‘ASA’ using a visual analog scale. They were on average less interested in ‘ChEIs’, ‘BRCA’ and ‘Prenatal’ topics (one-way ANOVA, p < 0.0001) (Figure 3). A significant difference was found in the degree of interest for clinical topics according to the type of clinicians (two-way ANOVA, p < 0.005): nurses were more interested in prenatal screening than physicians (P = 0.002) or residents (p = 0.002), and they were less interested in the PSA test than physicians (P = 0.0005) or residents (p = 0.008). Clinicians reported a level of satisfaction with the DBoxes of 4 or 5 on a 5-point smiley-faces rating scale ranging from 1 (sad face) to 5 (smiling face) in 81% of questionnaires completed (373/463; 33 missing responses). They were more satisfied with the ‘Prenatal’ DBox and least satisfied with the ‘OSTEO’ DBox (Table 2).

**Table 2:** **Mean level of satisfaction with each decision box of participating clinicians measured with a 5-point smiley-faces rating scale ranging from 1 (sad face) to 5 (smiling face)**

**Decision box clinical topics (abbreviations**)	**Mean level of satisfaction, range 1–5, 5 indicating higher satisfaction (± SD)**	**n**
The serum integrated test to screen women for fetal trisomy 21 *(Prenatal)*	**4.40 (0.85)**	**67**
The prostate-specific antigen test to screen men for prostate cancer *(PSA)*	**4.18 (0.87)**	**51**
Statins for primary prevention of cardiovascular disease *(Statins)*	**4.15 (0.78)**	**56**
Acetylsalicylic acid for primary prevention of cardiovascular disease *(ASA)*	**4.08 (0.94)**	**67**
The fecal occult blood test to screen for colorectal cancer *(FOBT)*	**4.07 (0.99)**	**61**
The BRCA1/2 gene mutation test to evaluate the risks of breast and ovarian cancer *(BRCA*)	**4.05 (0.94)**	**63**
Cholinesterase inhibitors to reduce the symptoms of Alzheimer’s disease *(ChEIs)*	**4.04 (0.99)**	**74**
Bisphosphonates to prevent osteoporotic fractures in postmenopausal women *(OSTEO)*	**3.98 (0.83)**	**57**

### Clinician’s questionnaire completion rate

**Figure 4:**
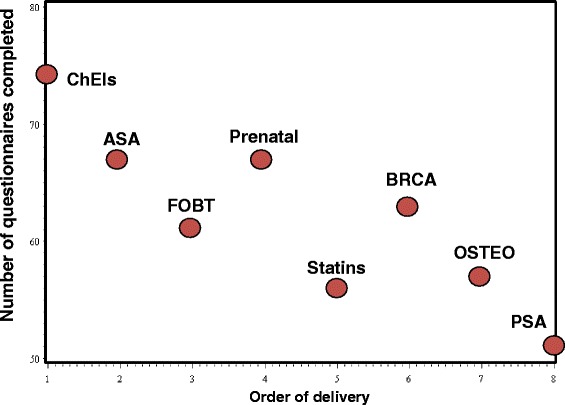
**Questionnaire completition rates.** Number of web-questionnaires completed by clinicians for each decision box according to its order of delivery (see Table 1 for the abbreviation legend).

Clinicians of the six participating clinics completed between four to seven of the eight web-questionnaires in each clinic and overall, 67% of clinicians completed at least 4 of the 8 questionnaires. In total, 808 questionnaires were sent to clinicians (eight per clinician, 101 clinicians), and of these, 61% were completed (496 responses out of 808 questionnaires) (Figure 2). There was an inverse relationship between the number of questionnaires completed for a DBox and its order of delivery: the first DBoxes sent were evaluated by more clinicians than the last ones (R^2^ = 0.74; P < 0.0001) (Figure 4).

### Patient screening, recruitment and eligibility rates

Of the 135 patients who were screened for eligibility across the four clinical sites, 37 (27%) were considered eligible to participate whereas 98 patients were not eligible because they did not discuss one of the eight DBox topics with their clinicians during consultation (Figure 2). Patients’ eligibility rate ranged from 9% to 53% depending on the participating clinic. Overall, we recruited 1–2 patients per clinic, per day. Thus, about seven days were required for a single attending clinician to discuss one of the targeted topics with a patient so that we could recruit one patient.

### Proportion of patient questionnaires completed by topic

Thirty-seven patients completed the questionnaire. The decision to use statins to prevent cardiovascular diseases was the most frequently discussed, with one third of patients who completed questionnaires based on this treatment (11 out of 37). Eighty-two percent of patients discussed the same four topics and none discussed cholinesterase inhibitors for Alzheimer’s disease (Table 3).

**Table 3:** **Frequency of patients who discussed each decision box topic in each participating primary healthcare clinic**

**Decision box clinical topics (abbreviations)**	**Clinics**				**Number patients**	**% patient (n = 37)**
	**1**	**2**	**3**	**4**		
Statins for primary prevention of cardiovascular disease *(Statins)*	0	1	7	3	**11**	**30**
The fecal occult blood test to screen for colorectal cancer *(FOBT)*	2	0	6	0	**8**	**22**
Bisphosphonates to prevent osteoporotic fractures in postmenopausal women *(OSTEO)*	1	1	3	1	**6**	**16**
Acetylsalicylic acid for primary prevention of cardiovascular disease *(ASA)*	2	0	3	0	**5**	**14**
The BRCA1/2 gene mutation test to evaluate the risks of breast and ovarian cancer *(BRCA*)	1	2	0	0	**3**	**8**
The serum integrated test to screen women for fetal trisomy 21 *(Prenatal)*	2	0	0	0	**2**	**5**
The prostate-specific antigen test to screen men for prostate cancer *(PSA)*	0	0	2	0	**2**	**5**
Cholinesterase inhibitors to reduce the symptoms of Alzheimer’s disease *(ChEIs)*	0	0	0	0	**0**	**0**

### Calculation of needed sample size

We are planning to use patients’ involvement in decision making during the consultation measured with the SDM-Q-9 [13] as primary outcome in the planned RCT. Based on the standard deviation for the SDM-Q-9 obtained in this feasibility study (18.5%), and on a previous study when we measured an ICC of 0.02 in a similar setting for a measure of the prescription of antibiotics after training of clinicians in SDM,[19] we estimated that a sample of 160 patients post-intervention would allow detecting a mean difference of 9% (corresponding to a conservative effect size of 0.5) between our two arms, considering a significance level of 0.05 and a power of 80%. This difference lies between the 3% mean difference measured in a systematic review of the impact of printed educational material on professional practice [16], and the 55% mean difference observed in another review on the impact of interventions tailored to the identified barriers for change [21]. We propose to use the same sample size pre-intervention, giving a total number of 320 patients in the planned RCT.

## Discussion

The purpose of this study was to assess the acceptability of the DBox to primary care clinicians, and the feasibility of an RCT to evaluate the impact of the DBox on patient’s involvement in decision-making during clinical consultation. The level of interest of participants for the DBox topics, and their level of satisfaction with the DBoxes support the acceptability of the intervention. Clinicians’ recruitment and questionnaire completion rates support the feasibility of the planned RCT, but clinics’ and patients’ recruitment processes should be optimized.

### Interpretation and implication of results

Clinicians’ level of satisfaction with the DBoxes was above the threshold that we set *a priori*, thus supporting the acceptability of DBoxes to participants. They were also generally interested in the selected DBox topics, thus validating that the Delphi panel that we used (described in [9]) was an appropriate strategy to select topics. We however found clinicians’ interest for the DBox topics differed across professions and topics, which might allow optimizing implementation of DBoxes in the next study by selecting the topics most appreciated for each profession.

The strategies to recruit the clinics in which we had no prior professional contact were inefficient. In contrast, we could easily recruit the clinics where we had prior professional contacts. Using personal contact and friendship networks is recognized as a powerful strategy to recruit study participants, but may not be feasible in a larger community and can lead to selection bias [22]. Hence, we plan to use an alternate strategy in the RCT, by inviting the 44 clinics that belong to practice-based research networks in Quebec. This strategy has previously been recommended based on empirical observations [23] and was successful in an earlier study in similar settings to implement a training program in SDM, in which recruitment rates reached 75% [24]. Using this strategy, we plan on recruiting 33 clinics in the next RCT.

The DBoxes take into account many of the factors influencing physician motivation to participate in training programs in SDM: they cover clinical topics perceived as relevant, are accessible, and include decision support tools [4]. This could account for the success of clinician recruitment that was above our 50%-criterion for feasibility of the planned RCT. To further improve clinician recruitment and response rates to the questionnaire, we plan to offer Continuing Professional Development (CPD) credits for each questionnaire completed as this can represent a significant incentive for some physicians [25,26].

Based on a study of a continuing medical education program in SDM, where 70% of the physicians attended at least two of the three workshops [19], we expected that about 70% of participating clinicians would complete at least half of the eight web-questionnaires. With a completion rate of 61%, we almost reached our target. This rate is relatively high compared to a study on research-based synopses delivered as daily email to 12 800 family physicians during which 1007 (8%) rated at least five of the synopses [27]. A systematic review of the methods to increase response rates to questionnaires showed that non-monetary incentives, such as entrance into a lottery, and a more interesting topic increased by more than a half the odds of response [28]. Hence, in the next study, we will deliver only the DBoxes covering topics perceived as more interesting, and that are more often discussed with patients. Clinicians had less interest in the topics covered in the BRCA, ChEIs and Prenatal DBoxes, and so these DBoxes will not be used in the planned RCT.

The patient recruitment rate was half of that expected, and this information will allow planning the resources required for the next study, by precisely estimating the length of the required patient recruitment period. To the best of our knowledge, there is no report on the rate of clinical decisions made in primary healthcare in Canada or elsewhere. A single study has presented 2-year prevalence of nine common medical decisions in a sample of 3,010 adults older than age forty from the US: three cancer screening decisions (for breast, colon, or prostate cancer); three long-term medications (for depression, high blood pressure, or elevated cholesterol); and three surgical procedures (for back pain, cataract, or hip/knee replacement) [29]. From this study, we unfortunately cannot infer the rate of these decisions in a given clinic, and we do not know if alternate decisions might be more prevalent than the one the authors chose to study. We observed in the present study that 82% patients discussed the same four topics and none discussed Cholinesterase inhibitors for Alzheimer’s disease. In the RCT, we plan to include the more frequently discussed topics, and consider adding topics frequently discussed in the US study cited above, such as depression. The inclusion of a larger number of clinics will ensure that the patient recruitment period will remain acceptable.

We observed an inverse relationship between the number of questionnaires completed for a DBox and its order of delivery. This indicates that we should randomize the order of delivery of the DBoxes among the clinics in the planned RCT. This also suggests that we should explore ways to sustain the interest throughout the intervention.

### Strengths and limitations of the study

This feasibility study provided clues to optimizing clinic and clinician recruitment and response rate to questionnaires for our planned RCT. Moreover, it allowed calculating the recruitment period needed to get the necessary patient sample size and thus plan the resources and budget.

This feasibility study was however limited in that its design differed from the design of the planned RCT: a single group of clinicians was exposed to the intervention, without any control group. Because of this, we could not test randomization methods and blinding of participants, nor estimate the effect of the intervention on the primary outcome. Convenience sampling also limits this study, since the participants may not have been representative of practicing clinicians and patients. A selection bias might have occurred as we were only able to recruit clinics where the study investigators had previous professional contacts. We should also have targeted a given patient sample size for each participating physician and not for each clinic, to ensure recruitment of patients seen by a diversity of physicians. Lastly, the small sample of nurses limits any results pertaining to them.

## Conclusion

In this feasibility study, no major challenges were identified regarding human and data management, or scientific issues. It provided data on the acceptability of DBoxes, and the feasibility of an RCT on the impact of the DBox on patient involvement in decision making processes, which will allow planning the sample size and resources needed for the next phase. Results from this feasibility study and from other pilot studies of educational trainings of clinicians to adopt SDM [19,30,31] may help inform other researchers address methodological challenges when planning a large RCT to evaluate shared decision making processes. This feasibility study also represents a case example of how to select outcome measures to evaluate the impact of SDM interventions using a conceptual framework based on goal settings activities proposed by Charles et al. [14]. Over the past three decades, the proportion of patients who prefer to participate in decision making during the clinical consultation has increased tremendously. The DBox answers a need expressed by patients and their families to be more involved in decision making. Furthermore, by training physicians in SDM, the DBox fosters an individualized approach to care, where patient preferences are taken into account when making a decision on treatment. The planned RCT will test the effectiveness of the DBox to increase patient involvement in decision-making, thus allowing a more judicious use of current best evidence in clinical decision making.

## Additional file

Additional file 1:
**Decision-making survey for patients.**
