# Supplementary material for: Feasibility of a randomized controlled trial to evaluate the impact of decision boxes on shared decision-making processes
Source: BMC Med Inform Decis Mak. 2015 Feb 25;15:13. doi: 10.1186/s12911-015-0134-x (PMC4350632; doi:10.1186/s12911-015-0134-x)
Supplement: Additional file 1: — Decision-making survey for patients. [file 12911_2015_134_MOESM1_ESM.pdf]

Decision-making survey for patients

1. **Please indicate which of the these health interventions was discussed with a clinic professional today:**

Taking **statins** to reduce your risks of cardiovascular disease	O
Prenatal screening for **trisomy** 21	O
Screening for **colon cancer**	O
Screening for prostate cancer	O
Taking aspirin to reduce your risks of cardiovascular disease	O
Taking medications to reduce your risks of fractures due to **osteoporosis**	O
Screening for mutations that increase the risk of having **breast and ovarian cancers**	O
Taking medications to treat **Alzheimer's** disease	O

2. If you discussed more than one of these interventions with the clinic professionals today, please indicate which you discussed the most:

____

Concerning this intervention that you discussed the most today, ...

3. With which healthcare professional did you discuss it?

Physician O	Nurse O	Other O<br>Please specify: ___________________

Nine statements related to the decision-making in your consultation are listed below. For each statement please indicate how much you agree or disagree.

Statement	completely disagree	strongly disagree	somewhat disagree	somewhat agree	stronglee agree	completely agree
**4. My doctor made clear that a decision needs to be made.**	☐	☐	☐	☐	☐	☐
**5. My doctor wanted to know exactly how I want to be involved in making the decision.**	☐	☐	☐	☐	☐	☐
**6. My doctor told me that there are different options concerning this intervention.**	☐	☐	☐	☐	☐	☐
**7. My doctor precisely explained the advantages and disadvantages of the options.**	☐	☐	☐	☐	☐	☐
**8. My doctor helped me understand all the information.**	☐	☐	☐	☐	☐	☐
**9. My doctor asked me which option I prefer concerning this intervention.**	☐	☐	☐	☐	☐	☐
**10. My doctor and I thoroughly weighed the different options concerning this intervention.**	☐	☐	☐	☐	☐	☐
**11. My doctor and I selected an option together.**	☐	☐	☐	☐	☐	☐
**12. My doctor and I reached an agreement on how to proceed.**	☐	☐	☐	☐	☐	☐

13. Please indicate which decision was made concerning this intervention (to take it, not to take it, to wait, to decide later, or other):

____

____

____

Still concerning the same intervention, please circle the answer of your choice:

Strongly agree	Agree	Neither agree nor disagree	Disagree	Strongly disagree

14. I know which options are available to me.

1	2	3	4	5

15. I know the benefits of each option.

1	2	3	4	5

16. I know the risks and side effects of each option.

1	2	3	4	5

17. I am clear about which benefits matter most to me.

1	2	3	4	5

18. I am clear about which risks and side effects matter most to me.

1	2	3	4	5

19. I am clear about which is more important to me (the benefits or the risks and side effects).

1	2	3	4	5

20. I have enough support from others to make a choice.

1	2	3	4	5

21. I am choosing without pressure from others.

1	2	3	4	5

22. I have enough advice to make a choice.

1	2	3	4	5

23. I am clear about the best choice for me.

1	2	3	4	5

24. I feel sure about what to choose.

1	2	3	4	5

25. This decision is easy for me to make.

1	2	3	4	5

26. I feel I have made an informed choice.

1	2	3	4	5

27. My decision shows what is important to me.

1	2	3	4	5

28. I expect to stick to my decision.

1	2	3	4	5

29. I am satisfied with my decision.

1	2	3	4	5

30. During your visit at the clinic, did you see any information concerning this decision?

Yes ○	No ○<br>Go to question #33

31. Did you see a Decision Box (please see the next page for an example)?

Yes ○	No ○<br>Go to question #33

32. Who showed you the Decision Box?

Physician ○	Nurse ○	Other ○<br>please describe: ___________________

Personal information

**33. Date of birth**	__________/_________/__________ (year/month/day)
**34. Employment status**	☐ Employed full-time<br>☐ Employed part-time<br>☐ Unemployed and seeking employment<br>☐ Unemployed and not seeking employment<br>☐ Retired<br>☐ Other, please specify ______________________________
**35. Highest education level**	☐ No high school<br>☐ Some high school - did not graduate<br>☐ High school degree or certificate of equivalency<br>☐ Some college – did not graduate<br>☐ College degree<br>☐ Some university – did not graduate<br>☐ University degree

Thank you for having completed this questionnaire
